# Predictors of response to ovulation induction using letrozole in women with polycystic ovary syndrome

**DOI:** 10.1186/s12902-023-01336-z

**Published:** 2023-04-25

**Authors:** Zaixin Guo, Shuwen Chen, Zhiyan Chen, Pan Hu, Yanfang Hao, Qi Yu

**Affiliations:** 1 Department of Obstetrics and Gynecology, National Clinical Research Center for Obstetric & Gynecologic Diseases, Peking Union Medical College Hospital, Peking Union Medical College and Chinese Academy of Medical Sciences, Beijing, China; 2 Department of Obstetrics and Gynecology, Peking University Shenzhen Hospital, Beijing, China

**Keywords:** Polycystic ovary syndrome, Letrozole, Ovulation

## Abstract

**Background:**

This study aimed to evaluate the predictive value of the initial screening characteristics of women with anovulatory polycystic ovary syndrome (PCOS) who did or did not respond to 2.5 mg letrozole (LET).

**Methods:**

The clinical and laboratory characteristics of women with PCOS who underwent LET treatment were evaluated. Women with PCOS were stratified according to their responses to LET (2.5 mg). The potential predictors of their responses to LET were estimated using logistic regression analysis.

**Results:**

Our retrospective study included 214 eligible patients with a response to 2.5 mg LET (n = 131) or no response to 2.5 mg LET (n = 83). PCOS patients who responded to 2.5 mg LET showed better outcomes than those who did not (2.5 mg LET) for pregnancy rate, live birth rate, pregnancy rate per patient, and live birth rate per patient. Logistic regression analyses showed that late menarche (odds ratio [OR], 1.79 [95% confidence intervals (CI), 1.22–2.64], *P* = 0.003), and increased anti-müllerian hormone (AMH) (OR, 1.12 [95% CI, 1.02–1.23], *P* = 0.02), baseline luteinizing hormone (LH)/ follicle stimulating hormone (FSH) (OR, 3.73 [95% CI, 2.12–6.64], *P* < 0.001), and free androgen index (FAI) (OR, 1.37 [95% CI, 1.16–1.64], *P* < 0.001) were associated with a higher possibility of no response to 2.5 mg LET.

**Conclusions:**

PCOS patients with an increased LH/FSH ratio, AMH, FAI, and late menarche may need an increased dosage of LET for a treatment response, which could be helpful in designing a personalized treatment strategy.

## Background

Polycystic ovary syndrome (PCOS) is a heterogeneous condition affecting approximately 20% of women worldwide and accounting for approximately 80% of cases of anovulatory infertility in women [1]. The mechanisms causing ovarian follicular arrest are complex, and the exact pathogenesis remains unknown [2, 3]. Ovulation induction is the first-line treatment among infertile women with PCOS, but the responses vary according to different ovarian stimulation protocols or different dosages of the same drug [2]. As personalized medicine, an advancing and accurate treatment, is expected, it is vital to identify the specific characteristics of PCOS patients and make accurate treatment plans.

Letrozole (LET), an aromatase inhibitor, has been recommended as a first-line therapy for anovulatory PCOS, which prevents the aromatase-induced conversion of androgens to estrogens, increases the secretion of follicle-stimulating hormone (FSH), and stimulates ovarian follicle development and maturation [4–6]. A meta-analysis showed that LET was better than clomiphene, the previous first-line agent, for ovulation rate per patient, pregnancy rate per patient, and live birth rate per patient [4, 5]. Also, LET resistance rates and multiple pregnancy rates appear lower with LET versus clomiphene [4, 5].

Usually, the starting dose of LET is 2.5 mg/day for 5 days (usually starting on day 3 of the cycle). The dose of LET should be increased to 5 mg and then 7.5 mg/day in subsequent cycles in cases of absent ovarian response. Using this approach, 49.4%~83.8% of patients ovulated in response to 2.5 mg LET [7, 8]. Higher dosage of LET may be needed for those un-responsive to 2.5 mg. Patients who ovulated with a higher dosage of LET would take longer to conceive and their compliance would be affected, especially for women of advanced age. Thus, predicting the possible doses of LET in different PCOS patients using their screening characteristics before ovulation induction may increase the effectiveness of treatment.

The objective of this study was to identify whether the pre-treatment characteristics reflecting the reproductive ability of PCOS patients had the predictive value for their ovarian response to the minimal ovulation doses of LET.

## Methods

### Study design and participants

This retrospective, single-center cohort study was approved by the Ethics Committee of Peking Union Medical College Hospital. Before the initiation of treatment, all patients had proven patency of at least one fallopian tube and normal semen analysis of their male partners. All patients who underwent ovulation induction with LET at Peking Union Medical College Hospital between April 2019 and July 2021 were evaluated for inclusion in the study. Eligible participants were women aged 20–38 years with a body mass index (BMI) ≤ 35 kg/m^2^, oligo-/anovulation, and a diagnosis of PCOS based on the Rotterdam consensus (two of three criteria: oligo-/anovulation, hyperandrogenemia, and sonographic appearance of polycystic ovaries). Diagnosis of oligo-/anovulation was defined as a menstrual cycle length > 35 days with < 8 menstrual cycles per year or no menstrual bleeding for 6 months or longer. Hyperandrogenemia was diagnosed either clinically (acne/hirsutism) or biochemically (testosterone ≥ 7.5ng/ml or free androgen index [FAI] ≥ 5). The ultrasound criteria included ≥ 12 follicles (2–9 mm) and/or ovarian volume > 10 ml. Patients with uncontrolled thyroid disease, hyperprolactinemia, adrenal hyperplasia, or Cushing’s syndrome were excluded.

### Assessment

Blood samples were drawn on days 2–4 of spontaneous or progesterone-induced menstruation. The following basal hormone assays were measured: follicle stimulating hormone (FSH), luteinizing hormone (LH), estradiol, total testosterone, dehydroepiandrosterone sulfate (DHEA-S), prolactin, sex hormone-binding globulin (SHBG), and anti-müllerian hormone (AMH) measured by specific immunoassays (Beckman kit, America).

### Interventions

Oral LET was prescribed orally daily for 5 days, starting on day 3 of the menstrual period or a progestogen-induced bleed. The starting dose of LET was 2.5 mg/day, and if pregnancy was not achieved, the dose was 5 mg/day in the second cycle. The maximum daily dose was 7.5 mg. LET resistance was defined as resistance to 7.5 mg LET for at least 1 cycle. A maximum of three or four cycles of ovulation induction was provided to the patients.

### Outcome parameters

Ovulation criteria were a follicle diameter ≥ 17 mm and ovulation monitored by ultrasound. Patients were stratified into two groups based on their response to LET (response to 2.5 mg LET or no response to 2.5 mg LET). All participants were advised about timed intercourse during the treatment cycles; couples were asked to refrain from intercourse until one follicle measuring at least 17 mm was found, and to keep sexual intercourse to every other day until ovulation. Live birth was defined as a live birth after ≥ 28 gestational weeks. Clinal pregnancy was defined as the presence of at least one gestational sac in the uterine cavity on ultrasonography at 5 weeks.

### Statistical methods

Continuous data were compared with the use of the Student’s t-test, and categorical variables were compared using the χ^2^ test. Variables were introduced into a multivariable logistic regression analysis in a stepwise fashion, with a univariate analysis (*P* < 0.30) to enter, and were retained in the multivariable model when the *P* value was < 0.05. Tables are presented with odds ratios (ORs) and corresponding 95% confidence intervals (CIs) for predictors in the adjusted logistic regression analysis. Cumulative probabilities for the outcomes of interest were determined using the Kaplan-Meier failure function (log-rank test) over four cycles according to stratified variables selected through multivariable logistic regression analysis. These variables were converted into dichotomous variables using receiver operating characteristic curves.

All data were analyzed using R (http://www.r-project.org), and a *P*-value < 0.05 was deemed statistically significant.

## Results

### Baseline data

A total of 214 eligible PCOS patients (605 cycles) were included in the analysis. A total of 131 (61.2%) patients ovulated with 2.5 mg LET, whereas 83 (38.8%) did not ovulate with LET (2.5 mg). Nine (4.2%) patients were LET resistant.

### Baseline characteristics

The baseline demographic, clinical, and endocrine characteristics are shown in Table 1. Patients who did not respond to LET (2.5 mg) had a late menarche compared to the 2.5 mg LET response group (mean [SD], 13.28 [1.29] vs. 13.69 [1.45] kg/m^2^, *P* = 0.042). Moreover, significantly higher serum AMH and baseline LH/FSH ratios were found in patients who did not respond to 2.5 mg LET (mean [SD]: 8.99 [4.94] vs. 10.52 [5.18] ng/ml, *P* = 0.042; 1.69 [0.87] vs. 2.09 [1.10], *P* = 0.005, respectively). The hyperandrogenemia indicators, including the modified Ferriman-Gallwey (mF-G) score, total testosterone, DHEA-S, and FAI, was not significantly different between the two groups.

**Table 1:** Characteristic of women who responded to 2.5 mg letrozole and who did not respond to 2.5 mg letrozole

Variables	Response to 2.5 mg letrozole (n = 131)	No response to 2.5 mg letrozole (n = 83)	P-value
Age, y (mean ± SD)	29.14 ± 3.18	28.61 ± 3.46	0.258
BMI, kg/m^2^ (mean ± SD)	23.09 ± 2.86	23.63 ± 3.10	0.197
Waist-hip ratio (mean ± SD)	0.84 ± 0.09	0.84 ± 0.06	0.557
Prior Gravidity, n (%)	37 (29.1)	21 (26.2)	0.771
Prior parity, n (%)	13 (10.2)	8 (10.0)	1.0
Menarche, y (mean ± SD)	13.28 ± 1.29	13.69 ± 1.45	**0.042**
mF-G score (mean ± SD)	3.82 ± 3.94	3.90 ± 3.59	0.886
AMH, ng/ml (mean ± SD)	8.99 ± 4.94	10.52 ± 5.18	**0.042**
Baseline FSH, IU/L (mean ± SD)	6.65 ± 1.84	6.89 ± 1.87	0.379
Baseline LH, IU/L (mean ± SD)	11.06 ± 6.03	13.88 ± 7.34	**0.004**
Baseline LH/FSH (mean ± SD)	1.69 ± 0.87	2.09 ± 1.10	**0.005**
Baseline E2, ng/mL (mean ± SD)	55.34 ± 35.10	54.16 ± 35.56	0.818
Baseline PRL, ng/mL (mean ± SD)	13.00 ± 6.91	11.35 ± 5.70	0.078
Total testosterone, ng/mL (mean ± SD)	0.69 ± 0.33	0.72 ± 0.28	0.521
SHGB, nmol/L (mean ± SD)	43.73 ± 32.79	41.71 ± 39.02	0.703
FAI (mean ± SD)	8.40 ± 8.20	10.34 ± 10.62	0.174
DHEA-S, ug/dL (mean ± SD)	270.88 ± 121.02	286.75 ± 135.42	0.412
HOMA-IR (mean ± SD)	2.64 ± 2.32	3.42 ± 3.80	0.075

*BMI* body mass index, *AMH* anti-müllerian hormone, *FSH* follicle stimulating hormone, *LH* luteinizing hormone, *E2* estradiol, *PRL* prolactin, *SHBG* sex hormone-binding globulin, *FAI* free androgen index, *DHEA-S* dehydroepiandrosterone sulfate, *HOMA-IR* homeostasis model assessment of insulin resistance

### Outcomes

Table 2 illustrates the outcomes, including the rates of pregnancy, live birth, and live birth per ovulating patient. In addition, the success rates per cycle, including pregnancy and live births, are shown. Pregnancy and live birth rates were significantly higher in the 2.5 mg LET response group (64.8% vs. 31.3%, *P* < 0.001; 52.7% vs. 21.7%, *P* < 0.001). The pregnancy and live birth rates per patient were also significantly higher in the 2.5 mg LET response group than in the other group (64.9% vs. 35.1%, *P* < 0.001; 52.7% vs. 24.3%, *P* < 0.001). Per cycle analysis revealed significantly higher pregnancy and live birth rates in the 2.5 mg LET response group (26.6% vs. 7.0%, *P* < 0.001; 21.6% vs. 6.3%, *P* < 0.001). Patients in the 2.5 mg LET no response group needed longer average cycles (mean [SD], 1.82 [0.90] vs. 2.77 [0.82], *P* < 0.001).

**Table 2:** Outcomes of women who responded to 2.5 mg letrozole and who did not respond to 2.5 mg letrozole

Variables	Response to 2.5 mg letrozole (n = 131)	No response to 2.5 mg letrozole (n = 83)	P-value
Pregnancy rate	85/131 (64.8%)	26/83 (31.3%)	**< 0.001**
Live birth rate	69/131 (52.7%)	18/83 (21.7%)	**< 0.001**
Pregnancies per ovulating patient	85/131 (64.9%)	26/74 (35.1%)	**< 0.001**
Live births per ovulating patient	69/131 (52.7%)	18/74 (24.3%)	**< 0.001**
Pregnancies per cycle	85/320 (26.6%)	26/285 (7.0%)	**< 0.001**
Live births per cycle	69/320 (21.6%)	18/285 (6.3%)	**< 0.001**
Average cycles taken to pregnancy (mean ± SD)	1.82 ± 0.90	2.77 ± 0.82	**< 0.001**

### Univariate and multivariate analysis

After adjusting for age (Table 3), logistic regression analyses showed that late menarche (odds ratio [OR], 1.79 [95% CI, 1.22–2.64], *P* = 0.003), AMH (OR, 1.12 [95% CI, 1.02–1.23], *P* = 0.02), baseline LH/FSH (OR, 3.73 [95% CI, 2.12–6.64], *P* < 0.001), and FAI (OR, 1.37 [95% CI, 1.16–1.64], *P* < 0.001) were correlated with a higher risk of no response to 2.5 mg LET.

**Table 3:** Univariate and multivariate regression analyses that compare variable clinical markers with respective outcomes

	Univariate*		Multivariate*	
Variables	OR (95% CI)	P-value	OR (95% CI)	P-value
Menarche	1.99 (1.45–2.75)	**< 0.001**	1.79 (1.22–2.64)	**0.003**
AMH	1.20 (1.10–1.30)	**< 0.001**	1.12 (1.02–1.23)	**0.02**
Baseline LH/FSH	3.02 (1.94–4.80)	**< 0.001**	3.73 (2.12–6.64)	**< 0.001**
FAI	1.41 (1.20–1.66)	**< 0.001**	1.37 (1.16–1.64)	**< 0.001**

*AMH* anti-müllerian hormone, *FSH* follicle stimulating hormone, *LH* luteinizing hormone, *FAI* free androgen index

*Adjusted for age

**Fig. 1:**
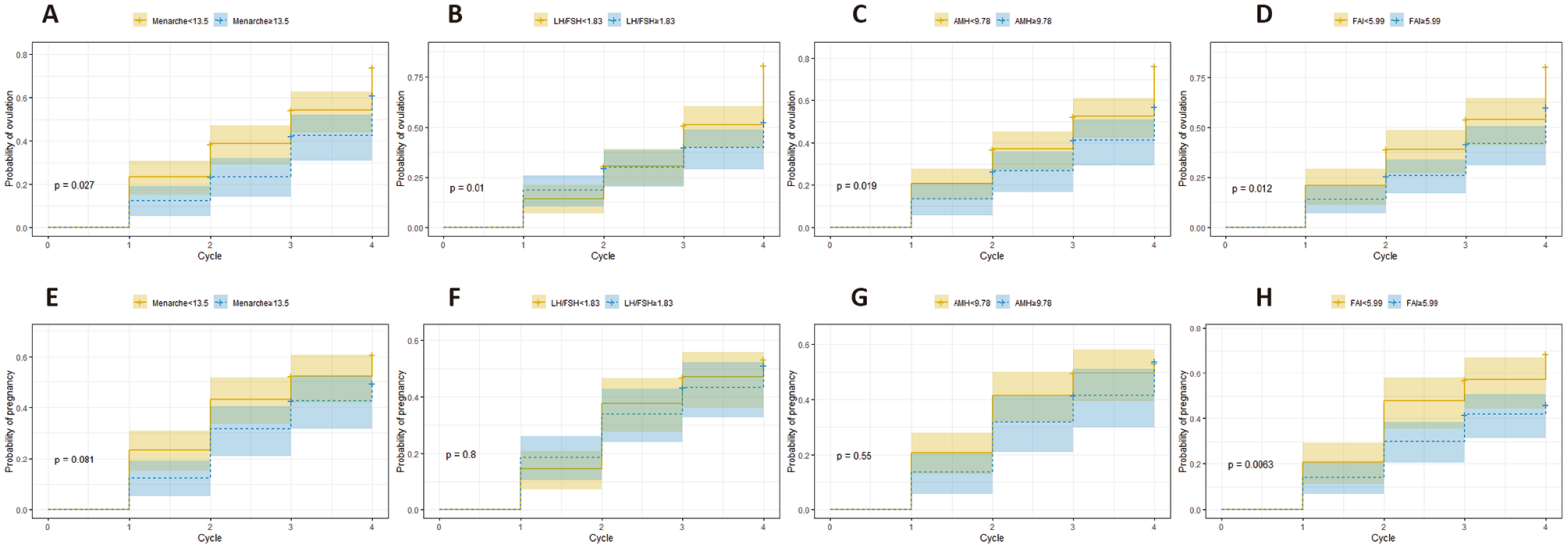
Unadjusted cumulative probabilities of achieving ovulation or pregnancy determined by the Kaplan-Meier failure function (log-rank test). (A) Probabilities of achieving ovulation, stratification on menarche < 13.5 years old and menarche ≥ 13.5 years old; (B) Probabilities of achieving ovulation, stratification on LH/FSH < 1.83 and LH/FSH ≥ 1.83; (C) Probabilities of achieving ovulation, and stratification on AMH < 9.78 ng/ml and AMH ≥ 9.78 ng/ml; (D) Probabilities of achieving ovulation, stratification on FAI < 5.99 and FAI ≥ 5.99; (E) Probabilities of achieving pregnancy, stratification on menarche < 13.5 years old and menarche ≥ 13.5 years old; (F) Probabilities of achieving pregnancy, stratification on LH/FSH < 1.83 and LH/FSH ≥ 1.83. (G) Probabilities of achieving pregnancy, stratification on AMH < 9.78 ng/ml and AMH ≥ 9.78 ng/ml; (H) Probabilities of achieving pregnancy, stratification on FAI < 5.99 and FAI ≥ 5.99; *AMH* anti-müllerian hormone, *FSH* follicle stimulating hormone, *LH* luteinizing hormone, *FAI* free androgen index

To further evaluate the influence of these indexes on fertility, we categorized the patients into two groups according to their menarche (menarche < 13.5y, menarche ≥ 13.5y), AMH (AMH < 9.78ng/ml, AMH ≥ 9.78 ng/ml), baseline LH/FSH (LH/FSH < 1.83, LH/FSH ≥ 1.83), and FAI (FAI < 5.99, FAI ≥ 5.99), and utilized Kaplan-Meier curves to describe ovulation and pregnancy in different groups. The cumulative ovulation rates of patients with menarche < 13.5y, LH/FSH ratio < 1.83, AMH < 9.78ng/ml, and FAI < 5.99 were significantly higher than that of patients with menarche ≥ 13.5, LH/FSH ratio ≥ 1.87, AMH ≥ 9.78 ng/ml, and FAI ≥ 5.99 (*P* = 0.027, 0.01, 0.019, and 0.012, respectively) (Fig. 1A-D). The cumulative pregnancy rate of patients with FAI < 5.99 was significantly higher than that of patients with FAI ≥ 5.99 (*P* = 0.0063) (Fig. 1H). The cumulative probabilities of pregnancy showed no significant differences between the menarche, LH/FSH, and AMH groups (Fig. 1E-G).

## Discussion

In the present study, we evaluated multiple characteristics associated with follicular responses to 2.5 mg LET doses in 214 patients with PCOS. Our results showed that late menarche, LH/FSH ratio, AMH and FAI were significantly higher in women with PCOS who did not respond to 2.5 mg LET. Cumulative ovulation rates were significantly lower in patients with menarche ≥ 13.5y, LH/FSH ratio ≥ 1.83, AMH ≥ 9.78 ng/ml, and FAI ≥ 5.99.

Fertility treatment in women with PCOS aims to restore monofollicular ovulation and achieve singleton pregnancy. “Low-tech” therapies, such as lifestyle modification and/or escalation of oral medication to achieve ovulation, are usually recommended [2]. Currently, LET represents the first line of treatment for patients with anovulatory infertility, whose conception and live birth rates can reach 41.2% and 27.5%, respectively; clomiphene, the previous first-line agent, provided conception and live birth rates of 27.4% and 19.1%, respectively [7]. Although the likelihood of live birth is increased by 40–60% with LET compared to clomiphene, the live birth rate is substantially lower than is generally assumed, which means it may take a relatively long time to search for help from assisted reproductive services if patients have lower chances of live birth with the LET protocol. Thus, it is necessary to describe the therapeutic effects of LET to help PCOS patients make better choices and improve their patience and compliance. In this study, we found that the dosage of LET-inducing ovulation could be a good index to discriminate women with PCOS who are expected to be pregnant with simple medical therapies. Our results showed that women who responded to LET (2.5 mg) had a significantly higher pregnancy rate (64.8%) and live birth rate (52.7%) than those who did not. Additionally, the chance of pregnancy was 26.6% per ovulatory cycle in women who responded to 2.5 mg LET, which was significantly higher than that in women who did not respond to 2.5 mg LET (7.0%), and similar to that in women without PCOS (10–15%) [9]. This means PCOS patients who respond to 2.5 mg LET are expected to have a similar chance of pregnancy with oral medication as those without PCOS. PCOS patients with no response to LET (2.5 mg) had a higher probability of seeking other complex therapies to get pregnant. The live birth rate was the most accurate index to present the effect of ovulation induction; however, the ovulatory responses of the minimal dosage of LET was also meaningful, acting as an easy and quick tool to partially reflect reproductive outcomes.

A higher baseline LH/FSH ratio has been shown to impair human reproduction. LH hypersecretion might cause premature luteinization of granulosa cells and increased production of androgens [10]. Relatively low FSH concentrations lead to inefficient aromatization of estrogen [11], which is detrimental to normal follicular growth. Previous studies have evaluated the importance of subgroups with high LH/FSH ratios for ovulation induction or assisted reproduction. An elevated baseline LH/FSH ratio is associated with poor ovulatory response but better clinical pregnancy and live birth after ovulation induction by clomiphene and/or acupuncture [12]. A basal LH/FSH ratio > 3 has an adverse effect on the number of follicles and oocytes, as well as on oocyte maturity in PCOS patients stimulated with human menopausal gonadotropins [13]. In addition, an LH/FSH ratio > 1.5 in PCOS patients who underwent in vitro maturation treatment led to a significant reduction in treatment [14]. An elevated LH/FSH ratio may influence the preferred protocol for PCOS treatment in in-vitro fertilization (IVF). PCOS patients with high LH/FSH ratios tended to have a higher probability of being pregnant using GnRH-agonist rather than GnRH-antagonist protocols [14], and this also affected the live-birth rate of fresh-embryo transfer cycle [15]. However, there are conflicting reports [16, 17]. Our results showed that the LH/FSH ratio was significantly higher in patients who did not respond to 2.5 mg LET and that LH/FSH ≥ 1.83 significantly impacted the success of ovulation induction by LET. This was different from previous results, probably due to the different populations, as the previous study only included a clomiphene-resistant population [18].

The elevated AMH level was another factor that profoundly affected the response to LET and proved to be related to fertility in PCOS. Hypersecretion of AMH in granulosa cells can impair follicular growth by inhibiting FSH and aromatase activity [19]. Previous results showed that serum AMH levels were significantly lower in cycles with a response to clomiphene than in cycles with no response [20–22]. Another study showed that PCOS patients with higher serum AMH levels have a lower possibility of response to clomiphene or LET [23]. Besides, high serum AMH levels are associated with a significantly lower probability of response to human menopausal gonadotrophin stimulation [24, 25]. High AMH is associated with lower live birth rates in women with PCOS undergoing assisted reproductive technology [26]. Our study also found a relationship between AMH levels and the effect of LET. The ovulation rate in patients with AMH < 9.78ng/ml was significantly higher than that in patients with AMH ≥ 9.78ng/ml. Therefore, we recommend that PCOS women with substantially elevated serum AMH levels induce ovulation with an increased dosage of LET.

An increased FAI also impacted the ovulatory responses to LET. Elevated androgen could inhibit ovarian follicular development, reduce oocyte meiotic capacity, and impact ovulation [27–29]. A previous study found that PCOS patients with a low hirsutism score had a higher possibility of conception, pregnancy, and live birth when ovulation was induced with clomiphene, metformin, or the combination of both [30]. PCOS patients with a lower total testosterone and higher SHBG concentrations also achieved pregnancy in a shorter time [31]. Similarly, hyperandrogenic PCOS phenotypes confer significantly lower cumulative live birth rates compared with their normo-androgenic counterparts who undergo IVF/intracytoplasmic sperm injection treatment [32]. Our study found that patients with FAI < 5.99 were associated with both higher cumulative ovulation rate and cumulative pregnancy when using LET. Thus, hyperandrogenism may be another indicator for using a higher dosage of LET to induce ovulation.

BMI was reported to impact ovulation but was not retained after stepwise selection in this study. BMI has been proven to reduce the reproductive outcomes including responsiveness to clomiphene, intrauterine insemination, and IVF [33–36]. Our research showed different results partially because few patients in our study were obese.

The major strength of our study is that we presented the ovulatory ability of LET by patient responses to a 2.5 mg dosage, which provides a different perspective to evaluate ovulation. Also, multiple characteristics associated with the follicular responses were evaluated which inspired us to develop personalized dosages of LET. Our study also had certain limitations. The sample size of this study was relatively small. This was a retrospective study; therefore, we could not provide an accurate model to predict the specific effects of LET in PCOS patients. In addition, we set the success of ovulation as the endpoint, which is a simple and direct index to evaluate the effect of LET, but could not represent the live birth rate.

## Conclusions

In conclusion, elevated LH/FSH, AMH, FAI, and late menarche are risk factors for poor ovulation induction in PCOS, which may requires a large than minimal dosage of LET.

## Data Availability

The datasets used in the current study are available from the corresponding author on reasonable request.

## Abbreviations

AMH: anti-müllerian hormone
BMI: body mass index
CI: confidence intervals
DHEA-S: dehydroepiandrosterone sulfate
FAI: free androgen index
FSH: follicle stimulating hormone
IVF: in vitro fertilization
LET: letrozole
LH: luteinizing hormone
mF-G: modified Ferriman-Gallwey
PCOS: polycystic ovary syndrome
OR: odds ratio
SHBG: sex hormone-binding globulin
